# Role of *Drosophila* EDEMs in the degradation of the alpha-1-antitrypsin Z variant

**DOI:** 10.3892/ijmm.2015.2109

**Published:** 2015-02-25

**Authors:** BO-YUN JANG, HYUNG DON RYOO, JAEKYOUNG SON, KYUNG-CHUL CHOI, DONG-MYOUNG SHIN, SANG-WOOK KANG, MIN-JI KANG

**Affiliations:** 1 Department of Biomedical Sciences, University of Ulsan College of Medicine, Seoul 138-736, Republic of Korea; 2 Cell Dysfunction Research Center and Bio-Medical Institute of Technology (BMIT), University of Ulsan College of Medicine, Seoul 138-736, Republic of Korea; 3 Department of Cell Biology, New York University School of Medicine, New York, NY 10016, USA

**Keywords:** endoplasmic reticulum stress, unfolded protein response, endoplasmic reticulum degradation-enhancing α-mannosidase-like protein, alpha-1-antitrypsin deficiency, Z variant of alpha-1-antitrypsin, null Hong Kong

## Abstract

The synthesis of proteins in the endoplasmic reticulum (ER) that exceeds the protein folding capacity of this organelle is a frequent cause of cellular dysfunction and disease. An example of such a disease is alpha-1-antitrypsin (A1AT) deficiency, caused by destabilizing mutations in this glycoprotein. It is considered that the mutant proteins are recognized in the ER by lectins and are subsequently degraded through the proteasome, leading to a deficiency in this enzyme in the afflicted patients. We previously established a *Drosophila* model of this disease by overexpressing the null Hong Kong (NHK) allele of this gene and found that the *Drosophila* lectin, ER degradation-enhancing α-mannosidase-like protein 2 (EDEM2), can accelerate the degradation of A1AT when overexpressed. NHK is a rare allele, and in this study, we investigated in depth the mechanisms through which *Drosophila* EDEMs affect the degradation of the Z variant, which is the predominant disease allele. Specifically, we report that the Z allele does not activate ER stress signaling as prominently as the NHK allele, but similarly requires both *Drosophila* EDEM1 and EDEM2 for the degradation of the protein. We demonstrate that EDEMs are required for their ubiquitination, and without EDEMs, glycosylated A1AT mutants accumulate in cells. These results support the role of the EDEM-mediated ubiquitination of the alpha-1-antitrypsin Z (ATZ) allele, and establish a *Drosophila* model for the study of this protein and disease.

## Introduction

**Figure 1:**
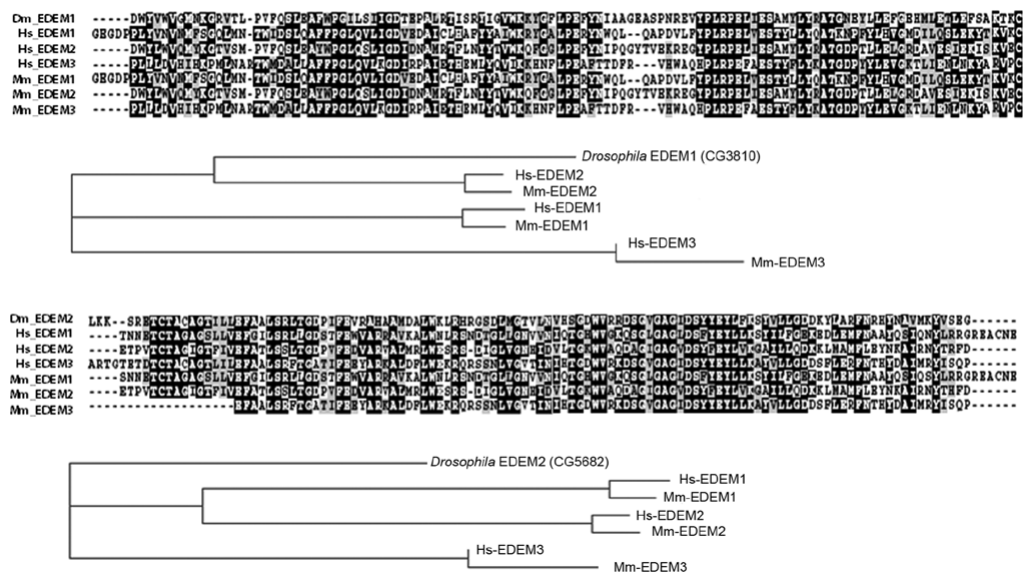
Alignment of the endoplasmic reticulum degradation-enhancing α-mannosidase-like protein (EDEM)1 and EDEM2 genes from *Drosophila* with the homologous proteins from human and mouse EDEM1-3. The intensity of darkness denotes the extent of conservation.

The endoplasmic reticulum (ER) is the cellular organelle in which membrane and secretory proteins are synthesized, glycosylated and acquire their correct conformation. While the properly folded proteins can leave the ER and traffick to their final destination along the secretary pathway, proteins that fail to fold are retrotranslocated to the cytoplasm for degradation, a process that is referred to as ER-associated degradation (ERAD) (1). ERAD is initiated by substrate recognition in the ER, followed by retrotranslocation into the cytoplasm for ubiquitin-mediated proteasomal degradation. For ERAD to occur properly, a machinery that can recognize misfolded proteins is required. While ER degradation-enhancing α-mannosidase-like proteins (EDEMs) were initially considered lectins (2,3), recent studies have revealed that EDEMs can function as mannosidases (4,5) and molecular chaperones (6). We have previously reported that the *Drosophila* genome encodes 2 EDEM homologs, EDEM1 (CG3810) and EDEM2 (CG5682) (14). Sequence analysis indicated that whereas *Drosophila* EDEM1 was similar to human EDEM2, *Drosophila* EDEM2 showed a closer resemblance to EDEM3 in mammals (Fig. 1).

Proteins that misfold in the ER underlie a number of conformational diseases in humans. Among these diseases is alpha-1-antitrypsin (A1AT) deficiency, which is caused by mutations in the A1AT gene that impairs its protein folding properties during biogenesis. The classical form of mutant A1AT protein is alpha-1-antitrypsin Z (ATZ) that results from a Glu342Lys substitution, rendering it prone to polymerization and aggregation (7). Misfolded ATZ is rapidly cleared from cells, through a combination of ERAD (8–10) and autophagy (11–13).

Previously, we established a *Drosophila* model to study how A1AT is degraded through ERAD (14). In that previous study, we had focused on the rare null Hong Kong (NHK) allele (2,3,15), and we had shown that the overexpression of *Drosophila* EDEM2 promotes ERAD of NHK. In this study, we performed a more in-depth investigation of *Drosophila* EDEMs, focusing on the predominant disease allele Z. Specifically, we demonstrate that the two *Drosophila* EDEMs play redundant roles in the degradation of the Z allele. We also demonstrate that the knockdown of these two genes leads to the accumulation of glycosylated ATZ proteins, while the overexpression of EDEMs promotes the degradation of ATZ. In addition, we provide evidence of A1AT ubiquitination, using cell-based ubiquitination assays.

## Materials and methods

### Plasmids and fly stocks

Genes were expressed in *Drosophila* through the standard GAL4/UAS system (16). The following flies and DNA have been described previously: armadillo-GAL4 (17), uas-myc-EDEM1 (14), uas-myc-EDEM2 (14) and uas-NHK (14). The DNA encoding ATZ (18) was subcloned into a pUAST plasmid.

### Cell culture and RNAi treatment

*Drosophila* Schneider 2 (S2) cells were grown in Schneider’s medium supplemented with 10% fetal bovine serum and 1% penicillin/streptomycin (Invitrogen, Grand Island, NY, USA). Treatment with double-stranded RNA (dsRNA) was performed as described in a previous study (19). Briefly, the cells were plated into 6-well plates at a density of 1×10^6^ cells/well before treatment with dsRNA (day 1). After 24 h, 20 *μ*g of EGFP, EDEM1 or EDEM2 dsRNA were added to each well following another boost with 20 *μ*g dsRNA on day 4. The cells were then transiently transfected with either NHK or ATZ using Effectene™ (Qiagen, Valencia, CA, USA) on day 5. The cells were split on day 8 and lysed to examine the level of NHK or ATZ on day 9. The EDEM1 dsRNA consisted of a 516-nt region (Amplicon ID: DRSC18573), as described by the *Drosophila* RNAi Screening Center (http://www.flyrnai.org). The following primers were used to amplify this sequence from an embryo cDNA library: ‘R’ primer, CAATGTTGTCACCCACGAAA; ‘S’ primer, TCGAAGTT GCTTACTAACAGA. This amplicon has no predicted off-targets. The EDEM2 dsRNA consisted of a 520-nucleotide region (amplicon ID: DRSC02877). The following primers were used to amplify this sequence from an embryo cDNA library: ‘R’ primer, 5′-CATGCGCGGGTTAAT CTC-3′; ‘S’ primer, 5′-GATAGAGCATCTCGTGTGTC-3′. To induce ER stress, *Drosophila* S2 cells were treated with dithiothreitol (DTT; Cat. no. 43815; Sigma, St. Louis, MO, USA) or thapsigargin (Tg; Cat. no. T9033; Sigma) for the indicated periods of time.

### Immunohistochemistry

All fluorescent images were captured under a Zeiss LSM 510 confocal microscope, using x100 objective lenses. The following antibodies were used: guinea pig anti-Hsc3 antibody (1:500) as previously described (17), mouse anti-myc (1:1,000 for immunohistochemistry; 9E10; Cat. no. 11667149001; Roche Diagnostics GmbH, Mannheim, Germany), rhodamine red anti-mouse secondary antibody (Cat. no. 715-295-150; 1:500) and FITC anti-guinea pig secondary antibody (Cat. no. 706-095-148; 1:500) (both from Jackson ImmunoResearch, West Grove, PA, USA).

### Western blot analysis and immunoprecipitation

For western blot analyses, *Drosophila* S2 cells were extracted with 1% SDS lysis buffer (10 mM Tris pH 7.5, 1 mM EDTA, 150 mM NaCl and 1% SDS; Sigma). Following centrifugation at 16,100 x g for 10 min, the supernatants were fractionated by SDS-PAGE and transferred onto polyvinylidene difluoride (PVDF) membranes (Millipore, Billerica, MA, USA). The membranes were then probed with the indicated antibodies: polyclonal rabbit anti-A1AT (1:5,000 for western blot analysis; Cat. no. A0012; Dako, Glostrup, Denmark), mouse anti-profilin (1:2,000 for western blot analysis; Developmental Studies Hybridoma Bank, chi 1J, University of Iowa, Iowa City, IA, USA), rat anti-HA antibody (Cat. no. 11867423001; 1:1,000; Roche Diagnostics GmbH) and rabbit anti-GFP antibody (1:5,000; Cat. no. A6455; Molecular Probes, Eugene, OR, USA). The fractionation of the *Drosophila* S2 cells was performed as previously described (20). For immunoprecipitation, the *Drosophila* S2 cells were extracted with 1% Triton X-100 lysis buffer (50 mM Tris pH 8.0, 150 mM NaCl, digitonin and 1% Triton X-100; Sigma) for 20 min on ice, and centrifuged at 16,100 × g. The supernatant was quantified by Bradford assay, and used for immunoprecipitation. Immunoprecipitation was performed with anti-A1AT antibody and protein-G-agarose beads (Roche Diagnostics GmbH). The beads were washed 3 times in low-ionic-strength buffer (50 mM Tris-Cl pH 8.0, 100 mM NaCl and 1% Triton X-100; Sigma). Rat anti-HA antibody was used to detect the ubiquitination of A1AT. For quantification of western bands, we used ImageJ software (http://rsbweb.nih.gov/ij). The intensity of the band of interest was normalized with an anti-profilin band.

### Measurement of RNA levels

Total RNA was isolated using TRIzol reagent, and reverse transcription was performed from 200 ng of total RNA using the SuperScript First-Strand Synthesis kit (both from Invitrogen). The following primer sequences were used for amplification and quantification: dEDEM1-F, ACGCCTACGATGGTTACCTG; dEDEM1-R, ACACGTTGATGTCCCTGTCA; dEDEM2-F, CTTAGCACCGAAACCACCAT; dEDEM2-R, ACTCCTCGGTACCGTCCTTT.

## Results

### Characterization of Drosophila EDEMs

**Figure 2:**
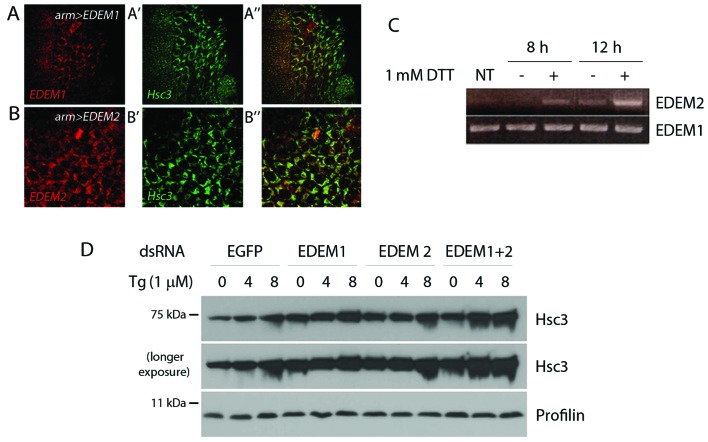
Characterization of *Drosophila* endoplasmic reticulum (ER) degradation-enhancing α-mannosidase-like proteins (EDEMs). (A and B) Subcellular localization of EDEMs. Immunohistochemistry of the embryonic amnioserosa cells revealed that myc-tagged EDEM protein (red) co-localizes with the ER resident chaperone Hsc3 (green). (C) Induction of EDEM2 expression by ER stress. *Drosophila* cells were treated with 1 mM dithiothreitol (DTT) for the indicated periods of time. The transcription of EDEM2, but not that of EDEM1, was induced by ER stress. NT, not treated (D) Hsc3 induction by thapsigargin (Tg) treatment increased after the knockdown of EDEM1 or EDEM2. The upper and middle panels show anti-Hsc3 western blots, whereas the lower panel shows anti-profilin as a loading control.

To determine the subcellular distribution of EDEMs in *Drosophila*, we generated transgenic EDEM lines with epitope tags and expressed them in *Drosophila* embryo amnioserosa cells. Immunolabeling with anti-myc antibody revealed that the *Drosophila* EDEMs co-localized with Hsc3, the *Drosophila* orthologue of mammalian BIP. This result is consistent with the hypothesis that *Drosophila* EDEMs reside in the ER (Fig. 2A and B).

Mammalian EDEMs are stress-regulated proteins that are induced by ER stress (21,22). To determine whether the *Drosophila* EDEM homologs are similarly regulated, we treated the *Drosophila* S2 cells with dithiothreitol (DTT), which imposes ER stress by reducing disulfide bonds. Under these conditions, *Drosophila* EDEM2 expression was transcriptionally induced (Fig. 2C). Similar results were obtained with independent ER stress-causing chemicals; tunicamycin (10 *μ*g/ml), which inhibits the glycosylation of proteins in the ER and thapsigargin (Tg; 1 *μ*M), which perturbs ER-calcium homeostasis (data not shown). On the other hand, we were not able to detect the induction of *Drosophila* EDEM1 under these conditions.

The degree of ER stress in cells can be indirectly assessed by the extent of the transcriptional response that induces ER chaperones and ERAD genes. To examine the protective role of *Drosophila* EDEMs under ER stress conditions, we treated the *Drosophila* S2 cells with dsRNAs that target either EGFP (as a control), EDEM1 or EDEM2, or both EDEM1 and EDEM2 and subsequently exposed them to Tg. The level of Hsc3 increased after 4 h, and this increase was even more pronounced when both EDEM1 and EDEM2 were knocked down (Fig. 2D). These results suggest that *Drosophila* EDEMs play protective roles against ER stress.

### The ER stress reporter is activated by NHK, but not by the ATZ variant

**Figure 3:**
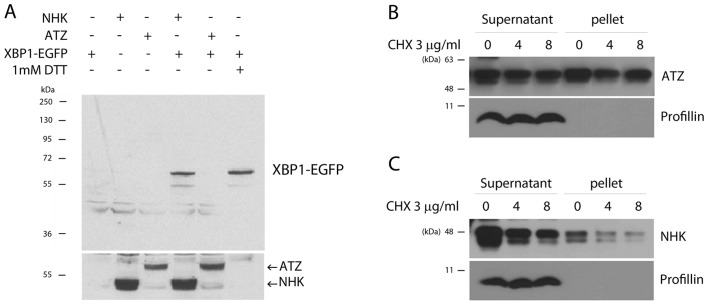
The Z variant of α-1-antitrypsin Ζ (ATZ) does not activate X-box binding protein 1 (XBP1)-EGFP, a marker of endoplasmic reticulum (ER) stress. (A) The XBP1-EGFP marker is activated by α-1-antitrypsin (A1AT) null Hong Kong (NHK) allele expression or by treatment with 1 mM dithiothreitol (DTT) treatment, but not by the expression of the ATZ allele. XBP1 splicing was evaluated by western blot analysis using anti-GFP antibodies (upper panel). The levels of ATZ and NHK expression were determined using anti-A1AT antibodies (lower panel). (B and C) Solubility of ATZ and NHK. *Drosophila* S2 cells transfected with ATZ or NHK were treated with cycloheximide (3 *μ*g/ml) for 0–8 h. The cells were separated into a supernatant and pellet fraction. (B) While western blot analysis with anti-A1AT antibody detected ATZ in both fractions, (C) almost all NHK was observed in the soluble (supernatant) fraction. Anti-profilin was used as a loading control.

Excessive protein misfolding in the ER triggers the activation of signaling pathways referred to as the unfolded protein response (UPR). One such pathway involves the mRNA splicing of X-box binding protein 1 (XBP1), which causes a shift in the reading frame of the downstream sequences and the generation of a distinct protein isoform (17,23). We have previously exploited this property to develop a UPR sensor, XBP1-EGFP, in which EGFP is expressed in frame when UPR is activated (17). We thus employed this UPR sensor to assess whether the mutant variants of A1AT cause ER stress in *Drosophila*. As we have reported previously (14), this UPR marker was activated by NHK expression (Fig. 3A, lane 4) to a similar extent as that induced by DTT treatment (Fig. 3A, lane 6). Intriguingly, ATZ expression in the *Drosophila* S2 cells did not trigger XBP1 mRNA splicing (Fig. 3A, lane 5). Previous studies on mammalian cells have also reported that, for some reason, ATZ does not activate UPR; instead, ATZ expression activates NF-κB signaling via ER overload response (EOR) and ERK signaling (24–26). To determine whether this difference is derived from the solubility of misfolded A1AT, we simply fractionated the cell extracts as supernatants and pellets. We found that the ratio of ATZ protein in the soluble versus the insoluble fraction was roughly 1:1 (Fig. 3B). On the other hand, the majority of NHK protein was found in the soluble fraction (Fig. 3C). These observations support the hypothesis that the two disease alleles of A1AT have distinct biochemical properties.

### The degradation of mutant variants of A1AT is regulated by Drosophila EDEMs

**Figure 4:**
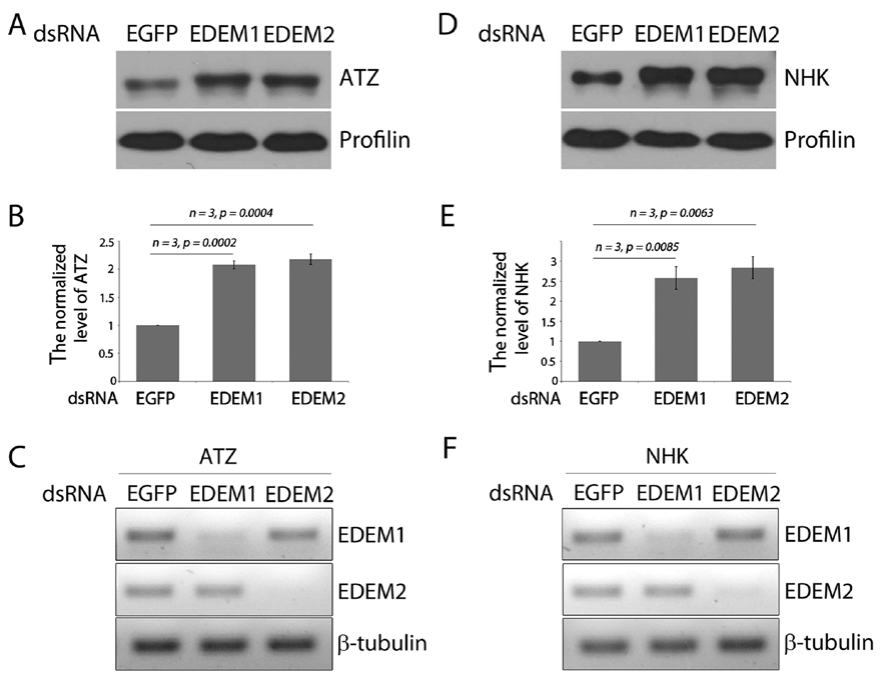
The knockdown of *Drosophila* endoplasmic reticulum (ER) degradation-enhancing α-mannosidase-like proteins (EDEMs) increases the level of mutant α-1-antitrypsin (A1AT) proteins. (A and D) The levels of the Z variant of α-1-antitrypsin Ζ (ATZ) and null Hong Kong (NHK) increased following treatment with dsRNAs that target *Drosophila* EDEM1 or EDEM2. (B and E) The fold change of misfolded A1AT expression level (n=3) was quantified using ImageJ software. The blot of profilin was used to normalize the signal of A1AT. Error bars represent the means ± SEM. (C and F) EDEM1 and EDEM2 mRNA levels were assessed by RT-PCR.

We have previously demonstrated that *Drosophila* EDEM1 and EDEM2 are homologs of mammalian EDEM2 and EDEM3, respectively. Moreover, we demonstrated that the overexpression of *Drosophila* EDEMs helps to lower the levels of the A1AT NHK variant (14). In this study, to investigate whether *Drosophila* EDEMs also show distinct specificity toward two misfolded A1AT variants, we examined the effects of EDEM1 and EDEM2 on the degradation of the misfolded ATZ and NHK variants. The level of ATZ increased by approximately 2-fold after the knockdown of EDEM1 and EDEM2 by dsRNA in *Drosophila* S2 cells (Fig. 4A and B). Similarly, the knockdown of *Drosophila* EDEM1 and EDEM2 increased the levels of another misfolded A1AT variant, NHK (Fig. 4D and E). Of note, a slightly higher molecular weight band of ATZ was detected after the knockdown of either *Drosophila* EDEM1 or EDEM2. As the deglycosylation of ERAD substrates occur after being dislocated back into the cytoplasm (27), the slower migrating A1AT band suggests defective ERAD and the accumulation of glycosylated ATZ species that accumulate in the ER.

**Figure 5:**
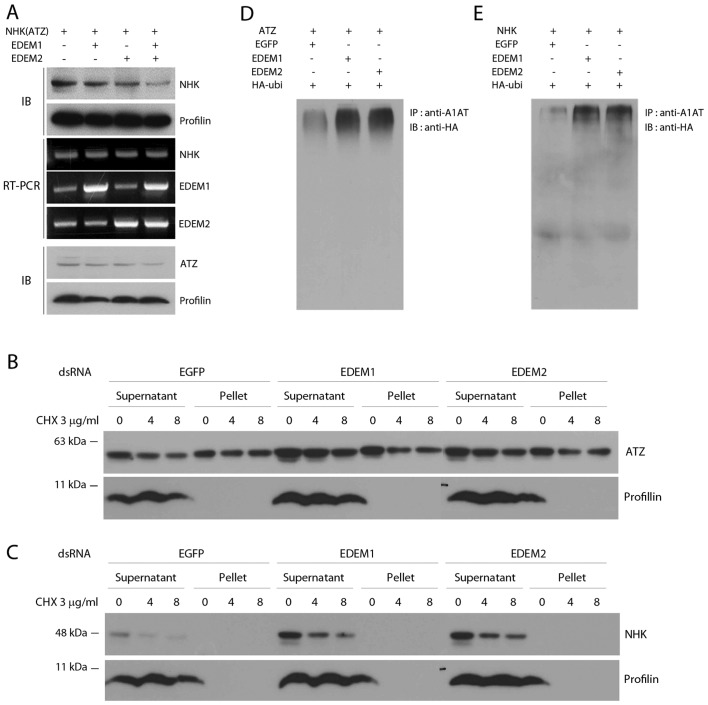
*Drosophila* endoplasmic reticulum (ER) degradation-enhancing α-mannosidase-like proteins (EDEMs) increase the degradation of misfolded α-1-antitrypsin (A1AT). (A) The A1AT variant was coexpressed with EDEM1 or EDEM2 in *Drosophila* S2 cells. A1AT transcript levels were assessed by RT-PCR, and protein levels were assessed by western blot analyses. (B and C) The solubility of α-1-antitrypsin Ζ (ATZ) or null Hong Kong (NHK) after the knockdown of *Drosophila* EDEM1 or EDEM2. *Drosophila* EDEMs did not have an obvious effect on the solubility of misfolded A1AT. The level of (B) ATZ or (C) NHK was analyzed in cells pre-treated with dsRNAs against EGFP (lanes 1–6), EDEM1 dsRNA (lanes 7–12), or EDEM2 (lanes 13–18). The lower gel shows anti-profilin bands as loading controls. (D and E) The ubiquitination of ATZ and NHK in transfected cells. *Drosophila* S2 cells were transfected with plasmids encoding HA-tagged ubiquitin and either ATZ or NHK together with *Drosophila* EDEM1 or EDEM2. (D) ATZ or (E) NHK was immunoprecipitated using anti-A1AT antibody, and the degree of ubiquitination of ATZ or NHK was analyzed by western blot analysis using the anti-HA antibody. IP, immunoprecipitation.

We also overexpressed EDEMs and found that mutant A1AT degradation was accelerated by the overexpression of EDEMs. Intriguingly, the two EDEMs from *Drosophila* had additive effects on the degradation of both the NHK (Fig. 5A) and ATZ variants of A1AT (Fig. 5A). Subsequently, we wished to determine whether *Drosophila* EDEM1 or EDEM2 affects the solubility of ATZ or NHK. We did not observe any significant change in the solubility of ATZ or NHK by knocking down EDEM1 or EDEM2 (Fig. 5B and C). These results indicate that *Drosophila* EDEMs regulate the degradation of misfolded A1AT variants without affecting the solubility of misfolded A1AT.

### Drosophila EDEMs increase the level of ubiquitinated misfolded A1AT variants

Previous studies have suggested that ATZ can be degraded by either the ubiquitin-proteasomal pathway, or through autophagy (13,28,29). In order to further confirm that the *Drosophila* EDEMs act by stimulating the ubiquitin proteasomal degradation of misfolded A1AT variants, we co-expressed *Drosophila* EDEMs and ATZ with HA-tagged ubiquitin. The co-expression of EDEM1 or EDEM2 with ATZ increased the level of ubiquitinated ATZ (Fig. 5D). The levels of ubiquitinated NHK also increased, albeit to a different extent than that observed for ATZ (Fig. 5E). These results again suggest that *Drosophila* EDEM1 and EDEM2 target misfolded A1AT variants for proteasomal degradation.

## Discussion

In the present study, we report the use of a *Drosophila* model for the study of the mechanisms of misfolded A1AT degradation that underly A1AT deficiency (30–32). Specifically, we focused on EDEMs, which are ER resident proteins with mannosidase-like domains. Similar to the mammalian EDEMs, we find that the *Drosophila* EDEM2 mRNA level increases in response to ER stress. We did not observe a similar induction with the EDEM1 mRNA level. The examination of the temporal and tissue-specific expression of *Drosophila* EDEM2 indicated that the tissues with the highest levels of *Drosophila* EDEM2 transcripts include the larval salivary gland, the adult intestine and the fat body, all of which have a high protein secretion load (33). Of note, these three organs are all characterized by high levels of IRE1/XBP1 activity (17,34). As mammalian EDEMs are regulated by IRE1/XBP1 signaling, it is likely that the *Drosophila* IRE1/XBP1 pathway contributes to the induction of EDEM2 during specific developmental stages, as well as upon ER stress.

EDEMs are involved in one of the early steps of ERAD substrate recognition. The tight regulation of ERAD is important as the inefficient detection of misfolded or unfolded proteins causes their accumulation in the ER, and leads to ER stress. On the other hand, overactive ERAD can degrade ER resident proteins or folding intermediates. Although the expression of the majority of ERAD components is upregulated by ER stress, we observed a significantly high level of *Drosophila* EDEM1 transcripts even under conditions of no stress (Fig. 2D). The mechanisms through which EDEM1 distinguishes terminally misfolded proteins versus folding intermediates remains to be explored.

In conclusion, the results from the present study indicate that the *Drosophila* EDEMs, EDEM1 and EDEM2, are resident in the ER, and that the expression of *Drosophila* EDEM2 is upregulated by ER stress. Both EDEM1 and EDEM2 in *Drosophila* promote the degradation of misfolded A1AT variants by increasing the ubiquitination of its substrates. Given the striking similarity between *Drosophila* and humans in terms of this process, the present study provides a novel approach for the study of A1AT deficiency.
